# Does Alcohol Cause Insanity?

**Published:** 1915-05

**Authors:** 


### ABSTRACTS

---

**Does Alcohol Cause Insanity?** H. Tyldesley, Central City, Ky., Ther. Rec., Mch. 1915, tabulates 200 cases of various types. 16 occurred in children of whom, 15 were total abstainers. 82 occurred in men, 22 total abstainers, 47 moderate and 13 excessive drinkers; 102 occurred in women, 79 total abstainers, 21 moderate and 2 excessive drinkers. Analysis of many of the cases in excessive drinkers seemed to show that alcohol had no very direct bearing on the mental state. (Some cases do not seem to have been strictly insane)..

